# Green entrepreneurial orientation, boundary-spanning search and enterprise sustainable performance: The moderating role of environmental dynamism

**DOI:** 10.3389/fpsyg.2022.978274

**Published:** 2022-10-17

**Authors:** Fang Ye, Yi Yang, Haiyan Xia, Yixuan Shao, Xieguo Gu, Jiaqiang Shen

**Affiliations:** ^1^College of Economics and Management, Zhejiang Ocean University, Zhoushan, China; ^2^College of Technology Integration, Kunsan National University, Gunsan, South Korea; ^3^Business School, Zhejiang Pharmaceutical Vocational University, Ningbo, China

**Keywords:** green entrepreneurial orientation, enterprise sustainable performance, boundary-spanning search, environmental dynamism, organizational evolution

## Abstract

As an important strategic decision for enterprise sustainability, the green entrepreneurial orientation can facilitate boundary-spanning search for external knowledge and resources to achieve triadic sustainable economic, environmental, and social performance. Based on organizational search theory and dynamic capability theory, this study introduces environmental dynamism into the model of the relationship between green entrepreneurial orientation, boundary-spanning search and enterprise triadic sustainable performance. By analyzing the questionnaire data from 202 managers of manufacturing SMEs, the study explores the internal and external influences of green entrepreneurial orientation on the enterprise sustainable performance. The results show that: green entrepreneurial orientation has a positive impact on enterprise economic, environmental and social performance; boundary-spanning search plays a fully mediating role between green entrepreneurial orientation and enterprise economic, environmental and social performance; environmental dynamism, as a key external environmental factor, positively regulates the relationship between boundary-spanning search and enterprise economic performance and environmental performance, and negatively regulates the relationship between boundary-spanning search and social performance. This study clearly demonstrates how green entrepreneurial orientation in the environmental era can drive triadic sustainable performance improvement of enterprises. In addition, this study argues that boundary-spanning search is an important tool that enables manufacturing SMEs to achieve a triad of coordinated sustainable development of economic, environmental and social benefits in a dynamic environment.

## Introduction

With the increasing prominence of environmental issues, sustainable development has emerged as the focus of global attention. 17 Sustainable Development Goals (SDGs) were adopted at the UN Sustainable Development Summit in September 2015, which aim to thoroughly address the three dimensions of development—social, economic and environmental—in an integrated manner and shift to a sustainable development path. In 2020, the 75th session of the UN General Assembly focused even more on global environmental issues and proposed action measures for the next phase of sustainable development. Leaders of major economies in April 2021 are focusing on the impact of the environment on climate change through climate summits, pushing major countries to reduce corporate pollution emissions. At the same time, green products are gradually becoming an important choice for consumers. For example, the electric vehicle market in Europe, North America, and Asia has been exploding in recent years, and “green electric vehicles” are gaining “traction” with consumers. In the new environmental era, driven by both government and the market, green entrepreneurship continues to emerge. Unlike traditional manufacturing companies, green entrepreneurial manufacturing companies focus on the organic combination of corporate profit pursuit and value creation to achieve the triple bottom line of sustainable development (economic development, social prosperity, and environmental friendliness; Elkington, 1998). Business managers have adopted green entrepreneurship as a strategic orientation to build green and sustainable business models that grow financially, improve the environment and provide social welfare through green innovation approaches (Alwakid et al., 2021). Therefore, in the new environmental era, it is significant to explore how green entrepreneurial orientation (GEO) can contribute to sustainable performance growth of manufacturing enterprises, especially SMEs, to address green innovation compensation, environmental and economic integration benefits.

What can be done to improve the sustainable performance of manufacturing SMEs? Researchers have conducted many meaningful explorations, focusing on both antecedents and boundary conditions. The antecedents are mainly reflected in the influence of entrepreneurs’ or managers’ traits, enterprise characteristics, organizational processes, and environmental improvements on the enterprise sustainable performance (ESP). For example, Ameer and Khan (2020) argues that younger managers can take a more holistic approach to sustainable practices in order to improve the environmental, social, and economic performance of their enterprises. Nor-Aishah et al. (2020) concluded that entrepreneurial leadership has a significant impact on environmental sustainability performance and social sustainability performance, but not on economic sustainability performance. Bourlakis et al. (2014) believed that firm size has a significant impact on economic performance, environmental performance but not social performance. Utami et al.’s (2019) study concluded that supply chain management practices have a significant and positive impact on the financial and environmental sustainability performance of SMEs in Indonesia. Yang et al. (2015) argued that in the manufacturing sector, supply chain integration has an important role in improving economic performance, but social performance shows an inverse effect. Compared with the antecedent exploration, boundary conditions focus more on the moderating effect of external factors such as industry environment, industry competition, market orientation, and government regulation on ESP. Porter (1991) states that industry structure plays a key role in capturing enterprise performance. In particular, industry power may influence indirect and direct competition among enterprises, which affects sustainable performance. Mukulu et al. (2011) suggest that socio-political support, regulation, business form organization and business development interventions are external factors that influence enterprise sustainability.

From the perspective of organizational evolution, sustainable development of enterprises is the pursuit of economic, environmental and social benefits in a unified process. The paradox of economic benefits, environmental costs, and social values often make the green transformation of manufacturing SMEs difficult to make decisions, and the inherent pursuit of sustainable performance of the three while balancing is a “pain point” for enterprises. Green entrepreneurial orientation, as a key factor that helps companies achieve sustainable performance, reflects the tendency of companies to integrate economic, environmental, and social benefits in their entrepreneurial activities (Habib et al., 2020). Green entrepreneurial orientation is the integration of the concept of green entrepreneurship into strategic orientation, the essence of which is to sustainably improve and enhance the quality of the ecological environment through individual or enterprise entrepreneurial activities, and ultimately achieve coordinated and sustainable development of financial performance, ecological environment and social responsibility (Wang et al., 2015b). The results of several researchers have verified the contribution and differential impact of GEO on financial performance (Jiang et al., 2018), environmental performance (Ndubisi and Nair, 2009), social performance (Masele, 2019), and innovation performance (Xia, 2019). Also, it has been concluded that green entrepreneurship (Extreme Green Entrepreneurship Leaders and Outsiders; Domańska et al., 2018), green entrepreneurial activity (the number of companies that have adopted environmental criteria as an indicator of green entrepreneurial activity; Alwakid et al., 2021) have a positive impact on the sustainability of the business triad, but they do not focus on the GEO. Therefore, from theoretical perspective, the GEO is conducive to promoting enterprise triadic sustainable performance, but the view that it can be balanced to achieve all three is rarely supported empirically (Habib et al., 2020). The more important unresolved issue is what impact the GEO may have on sustainable triadic performance of manufacturing SMEs in the environmental era and how the severe environmental turbulence that accompanies such states should be accommodated?

Further, the translation of the GEO into ESP depends on the external knowledge and resource acquisition of the top management of the enterprise. The acquisition of external heterogeneous knowledge and resources is an effective way for organizations to update existing practices and norms and enhance innovation capabilities (Ancona and Caldwell, 1990). Existing studies have focused more on the integration of existing knowledge and internal resources and neglected the development of external knowledge and resources (Yang et al., 2020). Boundary-spanning search (BSS) is a resource search behavior of enterprises across organizational or technological boundaries that can have a significant impact on sustainable acquisition performance (Jung and Lee, 2016; Wang et al., 2020a,b,c; Yang et al., 2021). Enterprise executives play a leading role in the selection of enterprise development paths, resource cultivation and integration, and market participation (Yang et al., 2020). Enterprises will give themselves a sustainable competitive advantage through BSS activities (Greve, 2007). Therefore, BSS is also considered as a special dynamic capability. Green entrepreneurial orientation, as a micro starting point of dynamic organizational competencies, plays a key role in the process of enterprise BSS. Enterprise executives rely on managerial GEO when they use BSS to learn, absorb and acquire heterogeneous knowledge and then implement strategic decisions. The innovative, environmental, pioneering and social green entrepreneurial orientation characteristics of enterprise executives can also influence specific boundary-spanning search behavior. The reason why the GEO described above has a differential effect on the economic, environmental and social triad of ESP is also closely related to BSS. The distinct reason is that for entrepreneurial orientation to translate into performance there must be actual managerial behavior, especially internal resource integration and technological breakthrough innovation, which requires enterprises to adopt BSS activities. There have been only a few studies exploring the mechanisms of GEO on the ESP (Habib et al., 2020), but no paper has focused on the mediating role of BSS behavior. At the same time, with increasing economic and environmental variables, environmental dynamism (ED) has become an important situational factor influencing the strategic decisions of top enterprise managers. Influenced by situational factors, enterprises perceive that changes in technology, market and demand dynamics affect their behavioral choices when making strategic decisions (Wang et al., 2015a). To adapt to such ED, enterprises search for available and unique resources beyond organizational or technological boundaries and convert such resources into resources that can create value for the enterprise, achieve technological breakthroughs, and accumulate sustainable competitive advantages. This shows that ED can influence GEO for ESP improvement at different stages.

According to the foregoing analysis, this study argues that ESP is the result of behavior that organically combines the development of the enterprise itself with value creation and pursues the triad of economic, environmental and social sustainability. Therefore, it is obvious that GEO plays an important role in the process of triadic sustainable acquisition performance. At the same time, in the environmental era, the determination of GEO by enterprise decision-makers needs to be supported by the organization’s ability to match BSS, and in the context of the ED of technology, market and demand, enterprise GEO drives BSS, which in turn affects economic, environmental and social triadic sustainable performance. Therefore, integrating the mediating role of BSS and the moderating role of ED, this study constructs the model of the acquisition process of enterprise triadic sustainable performance from GEO, and examines the influence paths and relationship patterns of GEO on enterprise triadic sustainable performance in the context of ED. Meanwhile, we select Chinese manufacturing SMEs as the research sample for empirical testing.

The remaining of the study is organized as follows. In the second section, this study presents the theoretical model and research hypotheses. In the third section, it describes the study design, including data collection, variables, and data processing. In the fourth section, the results of the analysis are reported, including common method bias tests, reliability and validity tests, descriptive statistics and correlations, and hypothesis testing. Finally, conclusions, theoretical implications and practical insights, and study limitations are discussed.

## Theoretical background and hypotheses

### Theoretical background

According to the research problems of this study, this study integrates the findings related to organizational search theory and dynamic capability theory to analyze the function mechanism and influence model of the relationship between GEO and ESP.

Firstly, the strategic development view of organizational search theory. Organizational search theory states that based on knowledge and technology search cost considerations, enterprises prefer local search early on to build competitive advantage (Rosenkopf and Nerkar, 2001). However, as enterprises grow and place greater demands on the organization for continuous innovation, they break through technological barriers or organizational boundaries to engage in search activities that cross boundaries. It has also been shown in the existing literature that the acquisition of sustainable competitive advantage relies heavily on the enterprise’s ability to search and reconfigure its knowledge beyond local boundaries (Kogut and Zander, 1992). Therefore, BSS is essentially a management behavior for enterprises to pursue sustainable development. Boundary-spanning search can provide rich heterogeneous resources for the organization to update its knowledge base, and identify and evaluate the external competitive environment. And timely adjustment of knowledge search activities according to environmental changes is an effective way to establish competitors’ imitation barriers and improve organizational performance (Miao and Wang, 2020). In terms of the strategic movement of organizational search, it is a strategic necessity for enterprises to conduct search across technological or organizational boundaries for continuous innovation, and the antecedent of search activities is a shift in the strategic orientation of the decision-makers themselves. Consequently, GEO is the logical starting point for enterprises’ BSS behavior, while ESP is the ultimate goal of BSS. Green entrepreneurial orientation with the goal of acquiring heterogeneous resources drives enterprises to break organizational practices as well as by acquiring knowledge and resources that are difficult for competitors to imitate. Thus, it accelerates the green product and service development process and promotes a more responsive response to market changes, which is an important strategic decision for sustainable acquisition performance.

Secondly, the organizational evolutionary view of dynamic capability theory. In a dynamically changing environment, an enterprise’s pre-existing knowledge and capabilities may become barriers to continuous innovation, and static capacity theory has difficulty explaining how enterprises gain competitive advantage in dynamic markets and why certain enterprises have sustained competitive advantage. In this context, Teece et al. (2016) introduced the concept of the ability to change capabilities namely dynamic capabilities. He defined dynamic capabilities as the ability of an enterprise to integrate, construct, and reconfigure internal and external resources to respond to a rapidly changing environment (Teece et al., 2016). Dynamic capabilities, as an intangible asset of the enterprise, coordinate the organization to continuously and repeatedly recombine resources to create new resource combinations that are valuable, scarce, unique, and difficult to imitate, driving the renewal of the organization’s strategy (Winter, 2003). Dynamic capabilities involve perception, capture, and transformation, while GEO includes green innovation, pioneering, environmentalism, and openness against risk. Thus, GEO seems to be related to the concept of dynamic capabilities (York and Venkataraman, 2010; Jiang et al., 2018). From a micro-source, dynamic capabilities are the reflection and evolution of strategic orientation at the organizational level, with the aim of better adapting to respond to changes in the external environment and maintaining competitive advantage (Rodenbach and Brettel, 2012). In the environmental era, the dynamic environment helps establish a strong GEO, providing new opportunities for enterprises to create, discover and exploit access to heterogeneous resources. It also motivates companies to move away from limiting their search for resources locally and to adopt resource search activities across technological or organizational boundaries. At the same time, uncertain technological and market environments force enterprises to break away from fixed management thinking and organizational practices, prompting managers to look at diverse external resources and match the innovative elements of enterprise resources with responses to changes in the external environment, which greatly increases the chances of breakthrough innovation and thus affects the acquisition of sustainable performance. Thus, it is clear that dynamic capabilities are a mechanism that influences enterprises to establish a GEO and conduct BSS to adapt to the requirements of the changing environment, which would provide a new explanation of the mechanism of acquiring and influencing the effect of ESP.

**Figure 1:**
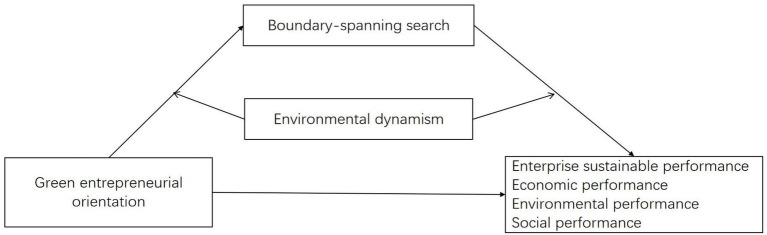
Theoretical model.

In sum, this study argues that GEO is a key influencing factor in the acquisition of ESP, while BSS behavior is the transmission mechanism for the relationship between the two. Therefore, this study constructs a conceptual model for GEO to obtain ESP through BSS, and focuses on the dynamic effect of ED. The model is shown in Figure 1.

### Research hypothesis

#### Green entrepreneurial orientation and enterprise sustainable performance

The involvement of manufacturing SMEs in sustainability management requires active decision-making by all managers or key decision-makers (Koe et al., 2014). Since the decision-making process is related to the cognitive process, the cognitive level of managers largely influences the decision-making. Green entrepreneurial orientation is a cognitive choice of top managers of enterprises and is considered as an important strategic decision for the sustainable development of enterprises (Pratono et al., 2019). Most of the available literature has concluded that GEO positively affects the acquisition of ESP (Koe et al., 2014; Jiang et al., 2018; Masele, 2019; Habib et al., 2020).

Green entrepreneurial orientation is a behavioral tendency and strategic posture that integrates entrepreneurial orientation with green value creation (Hughes et al., 2018), focusing on the strategic trends of enterprises to obtain economic, environmental, and social performance. The existing literature has focused on the relationship between GEO and financial performance, environmental performance, and social performance, respectively, in addition to fruitful studies on GEO and ESP. Magaji et al. (2017) showed that the innovative, Pioneering, risk-taking and competitive aggressive characteristics of GEO of manufacturing SMEs in Nigeria have a positive impact on financial performance. Makhloufi et al. (2020) described that green entrepreneurial oriented SMEs promote green Innovation by seeking to coordinate external environmental resources to innovate on environmental performance. Zhuang et al. (2020) concluded that GEO such as innovative, proactive, environmental, and social orientations of enterprises will lead them to adopt more socially responsible practices and bring benefits to society. Some studies have also focused on the dichotomous balance of GEO on economic and environmental performance, and environmental and socially responsible performance. For example, Habib et al. (2020) study concluded that GEO has a positive effect on economic performance and environmental performance through green supply chain management practices. Shafique et al. (2021) study argued that embedding socially responsible activities in enterprise strategies helps enterprises to play the role of GEO in order to achieve corporate social responsibility performance and environmental performance.

Therefore, theoretically, the GEO helps to promote triadic sustainable performance improvement in the manufacturing SME. Based on the above conclusions, we can derive an interesting transformation logic. Green entrepreneurial orientation provides SMEs with dynamic capabilities that enable these enterprises to explore, identify and evaluate resources that are closely related to market failures (Teece, 2012), providing strategic directions for enterprises to achieve economic benefits, environmental benefits and social welfare. It is evident that green entrepreneurial oriented enterprises need to find a balance of the three and valuable resources to achieve economic, environmental, and social performance alignment. Therefore, this study proposes the following hypothesis:

*H1a*: Green entrepreneurial orientation has a positive impact on the enterprise economic performance.

*H1b*: Green entrepreneurial orientation has a positive impact on enterprise environmental performance.

*H1c*: Green entrepreneurial orientation has a positive impact on enterprise social performance.

#### Green entrepreneurial orientation and boundary-spanning search

There is a direct relationship between the managerial behavior of enterprises and entrepreneurial orientation (Cordano and Frieze, 2000). Some study argues that executives with a strong entrepreneurial orientation are more likely to engage in entrepreneurial behaviors that involve knowledge acquisition across industries (Lichtenthaler, 2017). There are also studies that suggest that the entrepreneurial orientation of enterprise managers has a positive impact on their ability to build informal knowledge acquisition across organizational boundaries (Schierjott et al., 2018). This kind of search activity across organizational or technological boundaries is called “boundary-spanning search” (Yang et al., 2021). Derived from organizational search theory, BSS is an important way for enterprises to search, integrate and utilize external heterogeneous knowledge and resources to solve their own dilemmas and enhance their competitive advantages (Rosenkopf and Nerkar, 2001; Lichtenthaler, 2017). Existing studies have confirmed that entrepreneurial orientation (Schierjott et al., 2018), and social entrepreneurial orientation (Halberstadt et al., 2021) have a positive impact on BSS, but no studies have focused on the relationship between GEO on BSS.

Green entrepreneurial orientation is considered to be a behavioral tendency that influences enterprises’ access to internal and external resources (Makhloufi et al., 2021). Among the essence of green entrepreneurial enterprises to identify market opportunities, green orientation can lead manufacturing SMEs to generate a large demand for heterogeneous resources needed for green creation, however, manufacturing SMEs have limited internal resources to meet the changing technological and market needs and thus need to cross technological categories or organizational boundaries to find valuable and scarce resources (Slevin and Terjesen, 2011). Firstly, the innovative and pioneering of GEO facilitates the acquisition of complementary or alternative technological knowledge from external sources (Sofka and Grimpe, 2010), and the spillover effects of this knowledge can generate diverse and heterogeneous resources to support the growth of the enterprise’s internal resources (Robert Baum and Wally, 2003). Secondly, the environmental and social of GEO also help enterprises discover new knowledge linkages and knowledge combinations and obtain more valuable and unique knowledge options for enterprises to carry out green innovation in the environmental era (Wu and Shanley, 2009), which facilitates knowledge restructuring and knowledge integration to create green value. In conclusion, with the green innovative, pioneering, environmental, and social elements of GEO (Covin and Miller, 2014), through BSS, enterprises are not only able to access heterogeneous resources that match their own values, but also assess and adapt to changes in the environment in the process. And timely adjustment of knowledge structure and action guidelines according to market demand, scientific and effective integration of organizational knowledge resources (Wang et al., 2020c), as well as the discovery of potential market opportunities. Therefore, this study proposes the following hypothesis.

*H2*: Green entrepreneurial orientation has a positive impact on boundary-spanning search.

#### The mediating role of boundary-spanning search

Access to resources and knowledge is vital for ESP (Belso-Martínez et al., 2011). Boundary-spanning search is an essential way to acquire external knowledge, which is a way for enterprises to search for novel and heterogeneous knowledge and resources across organizational or technological boundaries to accelerate technological innovation and generate innovative knowledge that is difficult for competitors to imitate (Wang et al., 2020c). Therefore, BSS has become a key driver of ESP (Wang et al., 2020b). Meanwhile, the entrepreneurial orientation and entrepreneurial attitude of enterprise managers have a positive impact on their acquisition of informal knowledge across organizational boundaries (Schierjott et al., 2018; Banerjee, 2021). Existing studies have also confirmed that GEO helps enterprises to clarify their strategic direction and search orientation, which helps to generate new knowledge, thus contributing to innovation performance (Inkinen, 2016; Banerjee, 2021). It is shown that BSS has a significant impact between GEO and enterprise performance.

In the era of environmental protection, manufacturing SMEs’ mission of balancing economic, environmental and social co-development requires them to acquire more heterogeneous resources. Compared with large enterprises, manufacturing SMEs have the problem of weak access to resources, and it is difficult to cope with the rapid and dynamic changes in technology and market with the integration of internal resources alone. Thus there is a need to conduct search across technological and organizational boundaries to obtain heterogeneous knowledge that is difficult to be imitated by competitors (Miao and Wang, 2020). Based on organizational search theory, this study argues that by BSS, enterprises can not only increase the stock of knowledge within the organization, but also integrate external knowledge with the existing knowledge inventory (Katila and Ahuja, 2002). By “creating collisions” to generate new ideas and technologies (Laursen and Salter, 2014; Rui and Lyytinen, 2019), discovering a portfolio of knowledge that has economic, environmental, and social value. Specifically, firstly, green entrepreneurial-oriented enterprises search for economic knowledge and resources (e.g., technical knowledge and market knowledge) across borders to break the inertia of thinking and achieve breakthrough technological innovation through knowledge combination (Robert J. Sternberg, 2001), so as to promote economic performance improvement. Secondly, green entrepreneurial-oriented enterprises search for environmental knowledge and resources (e.g., green technologies, environmental management processes, and environmental standards) to help enterprises understand consumer green needs and government environmental regulatory requirements, thus promoting environmental performance improvement. Third, green entrepreneurial-oriented enterprises search for social knowledge and resources (e.g., community environment, social welfare) across borders, which helps enterprises organically combine their own values with social values, enhances their social reputation, shapes their social brand, and thus promotes social performance. Therefore, BSS provides a heterogeneous resource base for the triadic sustainable acquisition performance of enterprise. Therefore, this study proposes the following hypothesis.

*H3a*: Boundary-spanning search mediates between green entrepreneurial orientation and enterprise economic performance;

*H3b*: Boundary-spanning search mediates between green entrepreneurial orientation and enterprise environmental performance;

*H3c*: Boundary-spanning search mediates between green entrepreneurial orientation and enterprise social performance.

#### The moderating role of environmental dynamism

In the era of environmental protection, technology and market demand are changing rapidly, manufacturing SMEs only continuously adjust their strategic orientation, break through the boundaries of technology and organization to carry out BSS, so as to achieve sustainable development of enterprises.

1. Green entrepreneurial-oriented enterprises must conduct search activities across technological and organizational boundaries based on the external environment in order to adapt to dynamic environmental changes. Boundary-spanning search is a behavior to identify opportunities and solve problems by crossing organizational or technological boundaries, searching for valuable knowledge information, constantly updating existing knowledge structures, and effectively responding to dynamic environmental changes (Grimpe and Sofka, 2009). Therefore, ED has an important influence on the strategic choice and BSS behavior of green entrepreneurial enterprises. On the one hand, in the era of environmental protection, due to both government and market pressures, manufacturing SMEs need to implement GEO strategies (Kollmann and Stockmann, 2010) as soon as possible in order to gain new competitive advantages in order to make timely and effective responses to environmental changes. Based on the external environment, green entrepreneurial-oriented enterprises maintain their GEO to match the external dynamic environment through BSS activities to compensate for their own resource and capability deficiencies. Van Doorn et al. (2010) argue that in a dynamic market environment, GEO can help enterprises overcome path dependence and organizational inertia and facilitate the exploration of new knowledge and solutions. Therefore, based on dynamic capability theory, the interaction between GEO and dynamic environment can facilitate enterprises’ search and identification of market opportunities. On the other hand, the green innovation, pioneering and risk-taking characteristics of GEO make it more effective in dynamic environments and more conducive for organizations to break through existing organizational practices and mental models to achieve the creation of new products and technologies (Luo and Huang, 2019). It can be seen that the interaction between GEO and dynamic environment promotes enterprises’ BSS to form new knowledge structures. Therefore, ED driven by GEO may play a more active role in facilitating enterprises to respond to dynamic environmental changes through BSS. Therefore, this study proposes the following hypothesis.

*H4*: Environmental dynamism positively moderates the relationship between green entrepreneurial orientation and boundary-spanning search.

2. Environmental dynamism has an important impact on BSS and the acquisition of ESP. The more ED is, the faster the technology, market and demand will be updated, and green entrepreneurial enterprises will have stronger incentives to conduct BSS to acquire more heterogeneous knowledge for green technology innovation in response to environmental changes (Lin and Li, 2013). Conversely, if the motivation to conduct BSS is significantly lower, it will weaken the impact of BSS on green technology innovation (Wang et al., 2020a). In the above process, ED affects BSS behavior, which in turn has a differential impact on enterprises’ economic, environmental, and social performance. From the perspective of pursuing economic performance, the increased competitive intensity and uncertainty in the external market enables SMEs to be more proactive in conducting external search activities related to innovation (Jaiyeoba, 2013), as well as enhancing the knowledge base for technological innovation. This leads to the economic performance by acquiring economic resource elements for enterprises to stay ahead of their rivals in the competition of dynamic markets. From the perspective of pursuing environmental benefits, when the original knowledge structure needs to be changed in the face of environmental era and technological changes, it is difficult to adapt the specific and narrow internal knowledge search to the highly dynamic market environment (Zhang et al., 2013), thus external green technological knowledge and environmental knowledge are needed. Consequently, in the environmental era, the interaction of high ED and BSS instead helps enterprises achieve environmental performance and economic performance. From the perspective of pursuing social benefits, in the era of environmental protection, enterprises need to focus on social responsibility fulfillment and entrepreneurship establishment and combine more of this dynamic change in social progress with boundary-spanning social heterogeneity knowledge acquisition. Moreover, they can seek socially sustainable business opportunities in the midst of uncertainty and win more customers for their enterprises with their good social reputation. Therefore, in the context of ED, BSS behavior can be differentially influenced by different benefit pursuits (Li and Huang, 2021). Therefore, this study proposes the following hypothesis.

*H5a*: Environmental dynamism has positively moderated the relationship between boundary-spanning search and enterprise economic performance.

*H5b*: Environmental dynamism positively regulates the relationship between boundary-spanning search and enterprise environmental performance.

*H5c*: Environmental dynamism positively regulates the relationship between boundary-spanning search and enterprise social performance.

## Research design

### Sample and data collection

The research subjects are mainly from manufacturing SMEs in metal and non-metal products, textile and apparel industry, food manufacturing (mainly seafood manufacturing), pharmaceutical and chemical manufacturing, and transportation equipment manufacturing (mainly automobile and ship repair). Manufacturing enterprises, especially those in Asian countries, face problems such as low technological innovation capacity, low management level, high environmental damage and lack of sustainable development goals (Ndubisi et al., 2021). Meanwhile, Asian countries, represented by China, are vigorously promoting environmental programs and policies to achieve carbon neutrality in a progressive manner. Therefore, in the era of carbon neutrality or environmental protection, it is especially important for Chinese manufacturing SMEs to establish a GEO and build a green sustainable business model.

The survey respondents are SMEs from four industrial manufacturing zones in China’s coastal provinces. Because our focus is on small to medium-sized businesses, according to China’s ***Statistical Method for Dividing Large, Medium, Small and Micro Enterprises (2017)***, we constrained our sample to firms with not more than 1,000 employees and an operating income of <400 million yuan. To avoid potential common method variance and inherent relevant problems, this study used a combination of interview and questionnaire methods based on the development of a research model. Before the formal survey, we conducted in-depth interviews with 10 SME managers to understand the reality of the variable relationships. During the questionnaire phase, we conducted the survey in three stages. In the first stage, five senior academic experts and eight enterprise executives familiar with green entrepreneurship and enterprise performance were selected to review and revise the questionnaire. In the second stage, 20 enterprise executives were randomly selected to conduct the preliminary questionnaire survey. Statistics found that Cronbach’s *α* was >0.7 for all first-order structures, which indicated that the questionnaire was reliable. In the third stage, 250 sample enterprise managers were selected for the survey. First, the project team contacted the local government to obtain all enterprise data, and then 250 enterprises were randomly selected as the sample. The local government department will publish the survey notice on the enterprise work contact network. The chairman, general manager or senior managers of the sample companies were identified as respondents. Secondly, 18 surveyors (who received two rounds of training beforehand) were divided into four groups and visited the sample companies from August to October 2020 to conduct the survey. The questionnaires were collected after 10 days of distribution. The questionnaires were collected on October 28, 2020, and then coded by the investigators.

A total of 202 valid questionnaires were collected in this survey, of which 67.3% (136) respondents were at the decision-making level of SMEs. Respondents’ time in the position was mainly concentrated in 1–4 years, accounting for 52.5% (106). 55.5% (112) of enterprises age are over 5 years old, and Enterprise size was mainly concentrated in small businesses, accounting for 54.9% (111). See Table 1.

**Table 1:** Distribution of respondent characteristics (*N* = 202).

Characteristics	Category	Frequency (*N*)	Percentage (%)
Gender	Men	136	67.3
	Women	66	32.7
Educational background	Junior college and below	107	53.0
	Bachelor’s degree	77	38.1
	Master’s degree	16	7.9
	Doctorate	2	1.0
Management position	Executives	66	32.7
	Senior executives	57	28.2
	Assistants	12	5.9
	General managers	13	6.5
	Executive directors/Directors/CEOs	54	26.7
Position time	<1 year	20	9.9
	1–4 years	106	52.5
	5–9 years	41	20.3
	More than 10 years	35	17.3
Enterprise age	<1 year	13	6.4
	1–4 years	77	38.1
	5–9 years	44	21.8
	More than 10 years	68	33.7
Enterprise size	Under 50 people	111	54.9
	50–99 people	39	19.3
	100–299 people	30	14.9
	300–499 people	16	7.9
	500–1,000 people	6	3.0

### Measures

Except for the control variables, all variables were measured on a Likert 5-point scale, with 1 indicating “fully disagree” and 5 indicating “fully agree.”

#### “Enterprise sustainable performance” questionnaire

The ESP Questionnaire by Habib et al. (2020) was used, which includes 3 dimensions of economic performance, environmental performance and social performance, with 15 questions, such as “Your company reduces energy costs,” “Your company reduces waste (water and/or solid) emissions,” all of which ask about the status of the ESP. The internal consistency coefficient of the questionnaire, Cronbach’s *α*, was 0.926. Among them, Cronbach’s *α* for economic performance is 0.876, Cronbach’s *α* for environmental performance is 0.872, and Cronbach’s *α* for social performance is 0.882.

#### “Green entrepreneurship orientation” questionnaire

The GEO questionnaire developed by Xia (2019) was used, which includes four dimensions, green innovation orientation, green initiative orientation, environmental orientation, and social orientation, with 12 questions. Questions such as “Your company’s green transformation of existing products or production lines are relatively strong” were set to ask managers’ opinions on the GEO of enterprises. The internal consistency coefficient of the questionnaire, Cronbach’s *α*, was 0.921.

#### “Boundary-spanning search” questionnaire

The BSS questionnaire developed by O'Cass et al. (2014) was used, including two dimensions of prospective BSS and following BSS, with eight questions. Questions such as “Identify new opportunities in new markets and new customer groups ahead of competitors” and “Follow competitors to improve the quality of existing products” were set. The internal consistency coefficient of the questionnaire, Cronbach’s *α*, was 0.746.

#### “Environmental dynamism” questionnaire

The ED questionnaire developed by Miller (1983) was used to measure technological dynamics, demand uncertainty, and environmental variability, respectively. Five questions were set, such as “The behavior of competitors in this industry is easy to predict, and it is difficult for products or services to become obsolete.” The internal consistency coefficient of the questionnaire, Cronbach’s *α*, was 0.769.

#### Control variables

Drawing on previous research results, enterprise characteristic variables such as enterprise size and enterprise age as well as demographic variables such as management position and position-time were introduced into the model as control variables to reduce the interference of the above elements on the research results.

### Data processing

SPSS 20.0 and AMOS 20.0 were used to analyze and process the collected data.

## Data analysis and results

### Normal distribution and common method variance test

Sample data conforming to the normal distribution is a prerequisite for estimation of the structural equation model (SEM) using the maximum likelihood method. If the absolute value of the skewness coefficient of the measurement scale is <3.0 and the absolute value of the kurtosis coefficient is <10.0, this indicates that the sample data obey normal distribution. Descriptive statistical analysis of the sample variables was performed using SPSS 20.0, and the results showed that the range of values of the sample skewness coefficient was [0.014, 1.174] and the range of values of the absolute value of the kurtosis coefficient was [0.061, 2.368], so the data collected in this study obeyed a normal distribution.

Consider the sensitivity that business managers may have to basic enterprise information, entrepreneurial orientation, and other content. To reduce the psychological defenses of the subjects, this study was conducted using an anonymous method of investigation and to test for possible common method variance tests. The results showed that the eigenvalues of nine factors were >1, with the value of variance explained by the first factor of the eigenvalue when unrotated being 35.116%, which is lower than the 40% criterion, indicating that the common method variance problem in this study is within the permissible range.

### Reliability and validity tests

Reliability represents the internal consistency, stability and clustering of the questionnaire. Cronbach’s *α* coefficient and combined reliability CR are generally used to test this. Validity represents the correctness and discrimination of the questionnaire. Convergent validity is generally tested using standardized factor loadings, AVE of the measurement items. As can be seen from Table 2, the Cronbach’s *α* coefficient and the combined reliability CR of each questionnaire in this study were >0.7, which indicates that the reliability of the questionnaires are good. Exploratory factor analysis was performed using SPSS 20.0, and the KMO value of the scale was 0.769, and the Bartlett’s sphere test approximate chi-square value was 344.439, with a significance (sig.) of 0.000, which indicates that the sample data are suitable for factor analysis. Secondly, principal component analysis was conducted, factor extraction was based on the principle of eigenvalues >1, and rotation was performed using maximum variance, resulting in nine factors with a cumulative variance explained of 62.63%, and the loadings of the measured entries on the corresponding factors were all >0.5, which indicates that the selected questionnaire can better reflect the research constructs of this study. Meanwhile, the average variance extracted AVE of each variable was >0.5, which indicated that the questionnaire has good convergent validity.

**Table 2:** Reliability and convergence validity tests.

Variable	Number of items	Minimum factor load	Cronbach’s *α*	CR	AVE
GEO	12	0.700	0.921	0.933	0.538
BSS	8	0.575	0.746	0.878	0.517
ED	5	0.501	0.769	0.860	0.562
ESP	15	0.672	0.926	0.940	0.514

GEO, Green entrepreneurial orientation; BSS, Boundary-spanning search; ED, Environmental dynamism; ESP, Enterprise sustainable performance. Same below.

Discriminant validity was tested by comparing the results of validation analyses of different factorial models. Validated factor analysis using SPSS 20.0 for four variables: GEO, BSS, ED, and ESP. Single-factor, different 3-factor and 4-factor models were constructed by combining adjacent factors among variables. By comparing the fit indices among the models, it was found that the fit effect of the 4-factor model (*χ*^2^/df = 2.432, TLI = 0.911, CFI = 0.914, RMSEA = 0.078) was significantly better than the other models and the correlation matching indices all met the requirements. This indicates good discriminant validity among the variables in this study. The results are shown in Table 3.

**Table 3:** Discriminant validity test.

Model	*χ* ^2^	df	*χ* ^2^/df	TLI	CFI	RMSEA	Model comparison test		
							Model comparison	Δ*χ* ^2^	Δdf
Benchmark model (GEO, BSS, ED, ESP)	1687.974	694	2.432	0.911	0.914	0.078			
Three-factor Model 1 (GEO + BSS, ED, ESP)	1856.127	697	2.663	0.885	0.896	0.090	2 vs. 1	168.153^***^	3
Three-factor Model 2 (GEO, BSS + ED, ESP)	1996.820	697	2.865	0.725	0.741	0.104	3 vs. 1	308.846^***^	3
Three-factor Model 3 (GEO + ED, BSS, ESP)	2099.791	697	3.013	0.714	0.712	0.105	4 vs. 1	412.051^***^	3
Three-factor Model 4 (GEO + ESP, BSS ED)	2277.956	697	3.268	0.688	0.692	0.112	5 vs. 1	589.982^***^	3
One-Factor Model (GEO + BSS + ED + ESP)	2729.592	700	3.900	0.621	0.613	0.128	6 vs. 1	1067.842^***^	6

“+” indicates merging between variables. ***p < 0.001.

### Correlation analysis

Descriptive statistical analysis and correlation analysis of core variables were conducted using SPSS 20.0 and the results are presented in Table 4. Green entrepreneurial orientation was significantly and positively correlated with BSS (*r* = 0.799, *p* < 0.001), ESP (*r* = 0.543, *p* < 0.001). The BSS was significantly and positively correlated with ESP (*r* = 0.621, *p* < 0.001). It provided initial support for the hypothesis testing of this study.

**Table 4:** Descriptive statistics and correlation analysis of variables.

Variable	M	SD	EA	CS	MP	PT	GEO	BSS	ED	ESP
EA	3.590	1.287	1							
ES	2.570	1.392	0.584^***^	1						
MP	2.660	1.619	−0.155^*^	−0.153^*^	1					
PT	3.050	1.265	0.676^***^	0.413^***^	0.159^*^	1				
GEO	4.033	0.562	0.113	0.189^**^	−0.048	0.076	1			
BSS	3.705	0.448	0.119	0.166^*^	−0.083	0.096	0.799^***^	1		
ED	1.729	0.481	0.05	0.172^*^	0.118	0.039	0.260^***^	0.289^***^	1	
ESP	3.830	0.587	0.228^**^	0.206^**^	−0.123	0.181^**^	0.543^***^	0.621^***^	0.385^***^	1

EA, Enterprise age; CS, Enterprise size; MP, Management position; PT, Position time.

*** *p* < 0.001;

** *p* < 0.01;

* *p* < 0.05 (double-tailed test).

### Hypothesis testing

#### Main effects test for stratified regression

Hierarchical regression was used to test the effect of GEO on enterprise triadic sustainability performance, and the results are presented in Table 5.

**Table 5:** Regression analysis results.

Model	EA	CS	MP	PT	GEO	BSS	ED	GEO × ED	BSS × ED	*R* ^2^	Δ*R*^2^	*F*
M_1_	−0.014	0.138	−0.074	0.060						0.033	0.014	1.696^***^
M_2_	−0.014	−0.008	−0.058	0.058	0.795^***^					0.643	0.634	70.659^***^
M_3_	−0.014	−0.027	−0.075	0.066	0.771^***^		0.100^**^			0.652	0.641	61.491^***^
M_4_	−0.034	−0.026	−0.028	0.091	0.775^***^		0.104^**^	0.163^**^		0.654	0.479	52.344^***^
M_5_	0.071	0.044	−0.193^*^	0.135						0.082	0.064	4.420^**^
M_6_	0.070	−0.020	−0.186^*^	0.134	0.350^***^					0.200	0.180	9.816^***^
M_7_	0.079	−0.008	−0.165^*^	0.112		0.379^***^				0.221	0.201	11.125^***^
M_8_	0.074	−0.018	−0.170	0.118	0.131	0.275^***^				0.227	0.152	9.555^***^
M_9_	0.031	−0.019	−0.206	0.143		0.344^***^	0.152^**^		0.119^**^	0.255	0.097	9.488^***^
M_10_	0.075	0.090	−0.131	0.075						0.061	0.042	3.198^**^
M_11_	0.074	−0.002	−0.122	0.074	0.496^***^					0.298	0.280	16.668^***^
M_12_	0.083	0.005	−0.086	0.039		0.609^***^				0.419	0.404	28.287^***^
M_13_	0.082	0.003	−0.088	0.040	0.033	0.582^***^				0.418	0.335	23.449^***^
M_14_	0.081	−0.037	−0.128	0.061		0.547^***^	0.219^**^		0.036^**^	0.461	0.182	23.675^***^
M_15_	0.080	0.136	0.085	0.030						0.048	0.028	2.461^*^
M_16_	0.080	0.037	0.096	0.029	0.339^***^					0.328	0.310	19.095^***^
M_17_	0.088	0.053	0.129^*^	−0.006		0.599^***^				0.394	0.379	25.540^***^
M_18_	0.086	0.040	0.123^*^	0.002	0.169	0.465^***^				0.405	0.307	22.092^***^
M_19_	0.070	0.005	0.071	0.029		0.342^***^	0.192^**^		−0.104^**^	0.468	0.198	24.341^***^

*** *p* < 0.001;

** *p* < 0.01;

* *p* < 0.05 (double-tailed test).

Compared to M5, M6 shows that GEO has a positive effect on enterprise economic performance after controlling for enterprise characteristics variables and demographic variables (*β* = 0.350, *p* < 0.001), and that GEO explains 18% of the variance in enterprise economic performance (Δ*R*^2^ = 0.180). Hypothesis H1a was accepted. Compared to M10, M11 indicates that GEO has a positive impact on ESP (*β* = 0.496, *p* < 0.001), and that GEO explains 28% of the variance in ESP (Δ*R*^2^ = 0.280). Hypothesis H1b was accepted. Compared to M15, M16 indicates that GEO has a positive impact on enterprise social performance (*β* = 0.539, *p* < 0.001), and that GEO explains 31% of the variance in enterprise social performance (Δ*R*^2^ = 0.310). Hypothesis H1c was accepted. According to the above analysis, GEO shows a significant correlation with economic performance, environmental performance, and social performance, and GEO also has a positive effect on overall ESP (*β* = 0.542, *p* < 0.001).

Further analysis, compared to M1, M2 showed that GEO has a positive effect on BSS in the case of control variables (*β* = 0.795, *p* < 0.001), and that GEO explained 33.4% of the variance in BSS (Δ*R*^2^ = 0.334). Therefore, H2 is accepted. Meanwhile, as shown by M6 and M8, the effect of GEO on economic performance ranges from significant (*β* = 0.350, *p* < 0.001) to insignificant (*β* = 0.131, *p* > 0.05) when GEO, BSS and enterprise economic performance are included in the model simultaneously. Moreover, as shown by M7, BSS has a positive effect on economic performance (*β* = 0.379, *p* < 0.001) and BSS explains 22.1% of the variance in economic performance (Δ*R*^2^ = 0.221). Thus, BSS has a fully mediated effect between GEO and economic performance. H3a is accepted. As shown by M11 and M13, the effect of GEO on environmental performance also ranged from significant (*β* = 0.496, *p* < 0.001) to insignificant (*β* = 0.033, *p* > 0.05) when green GEO, BSS and enterprise environmental performance were included in the model simultaneously. Moreover, as shown by M12, BSS has a positive effect on environmental performance (*β* = 0.609, *p* < 0.001), and BSS explains 41.9% of the variance in economic performance (Δ*R*2 = 0.419). Thus, BSS has a fully mediating effect between GEO and environmental performance. H3b is accepted. M16 and M18 show that the effect of GEO on environmental performance also ranges from significant (*β* = 0.339, *p* < 0.001) to insignificant (*β* = 0.169, *p* > 0.05) when GEO, BSS and enterprise social performance are included in the model simultaneously. In addition, as shown by M17, BSS has a positive impact on social performance (*β* = 0.599, *p* < 0.001) and BSS explains 39.4% of the variance in economic performance (Δ*R*^2^ = 0.394). Thus, BSS has a fully mediating effect between GEO and social performance, and H3c is accepted.

#### Mediating effects test for bootstrap

To further test the analysis, with the help of the Process program in SPSS 20.0, this study use the Bootstrap method proposed by Preacher and Hayes (2004) to verify the mediating effect of BSS. The sample size of Bootstrap was set to 5,000, and the results are shown in Table 6.

**Table 6:** Bootstrap analysis results.

Path Relationships	Direct effect	Bootstrap 95%CI		Indirect effects	Bootstrap 95%CI		Total effect	Bootstrap 95%CI	
		LLCI	ULCI		LLCI	ULCI		LLCI	ULCI
GEO → BSS → ECP	0.131 (0.105)	−0.077	0.339	0.219 (0.092)	0.041	0.408	0.350 (0.065)	0.221	0.478
GEO → BSS → ENP	0.033 (0.092)	−0.148	0.213	0.464 (0.077)	0.314	0.622	0.497 (0.061)	0.376	0.616
GEO → BSS → SOP	0.169 (0.093)	−0.014	0.352	0.370 (0.072)	0.228	0.515	0.539 (0.060)	0.421	0.657

“()” is a standard error; in addition, ECP, Economic performance; ENP, Environmental performance; SOP, Social performance.

As can be seen from Table 6, the direct effect value of BSS between GEO and economic performance using Bootstrap test is 0.131, with 95% CI of [−0.077, 0.339], which does containing 0. This indicates that the effect of GEO on economic performance is not significant after the inclusion of BSS, controlling for enterprise characteristics variables and demographic variables. Meanwhile, the indirect effect value of BSS is 0.219, with 95% CI of [0.041, 0.408], which does not contain 0. H3a is accepted. In addition, the direct effect compared with the indirect effect shows that BSS plays a fully mediating effect between GEO and economic performance.

The direct effect value of BSS between GEO and environmental performance was 0.033, with 95% CI [−0.148, 0.213], which does contain 0. This indicates that the effect of GEO on environmental performance was not significant after the inclusion of BSS. Meanwhile, the indirect effect value of BSS was 0.464, with 95% CI [0.314, 0.622], which does not contain 0. H3b was accepted. Moreover, the direct effect compared with the indirect effect indicates that BSS plays a full-complete mediating effect between GEO and environmental performance.

The direct effect value of BSS between GEO and social performance is 0.169, with 95% CI [−0.014, 0.352], which does contain 0. This indicates that the effect of GEO on environmental performance is not significant after the inclusion of BSS. Meanwhile, the indirect effect value of BSS is 0.370 with 95% CI of [0.228, 0.515], which does not contain 0. H3c is accepted. In addition, the comparison of direct and indirect effects showed that BSS plays a full-complete mediating effect between GEO and social performance.

The above findings further verify the full mediating effect of BSS in GEO on economic performance, environmental performance, and social performance.

#### Test for moderating effect of multiple regression

Controlling for enterprise characteristic variables and demographic variables, this paper uses regression analysis to verify the moderating effect of ED, and the results are shown in Table 5.

**Figure 2:**
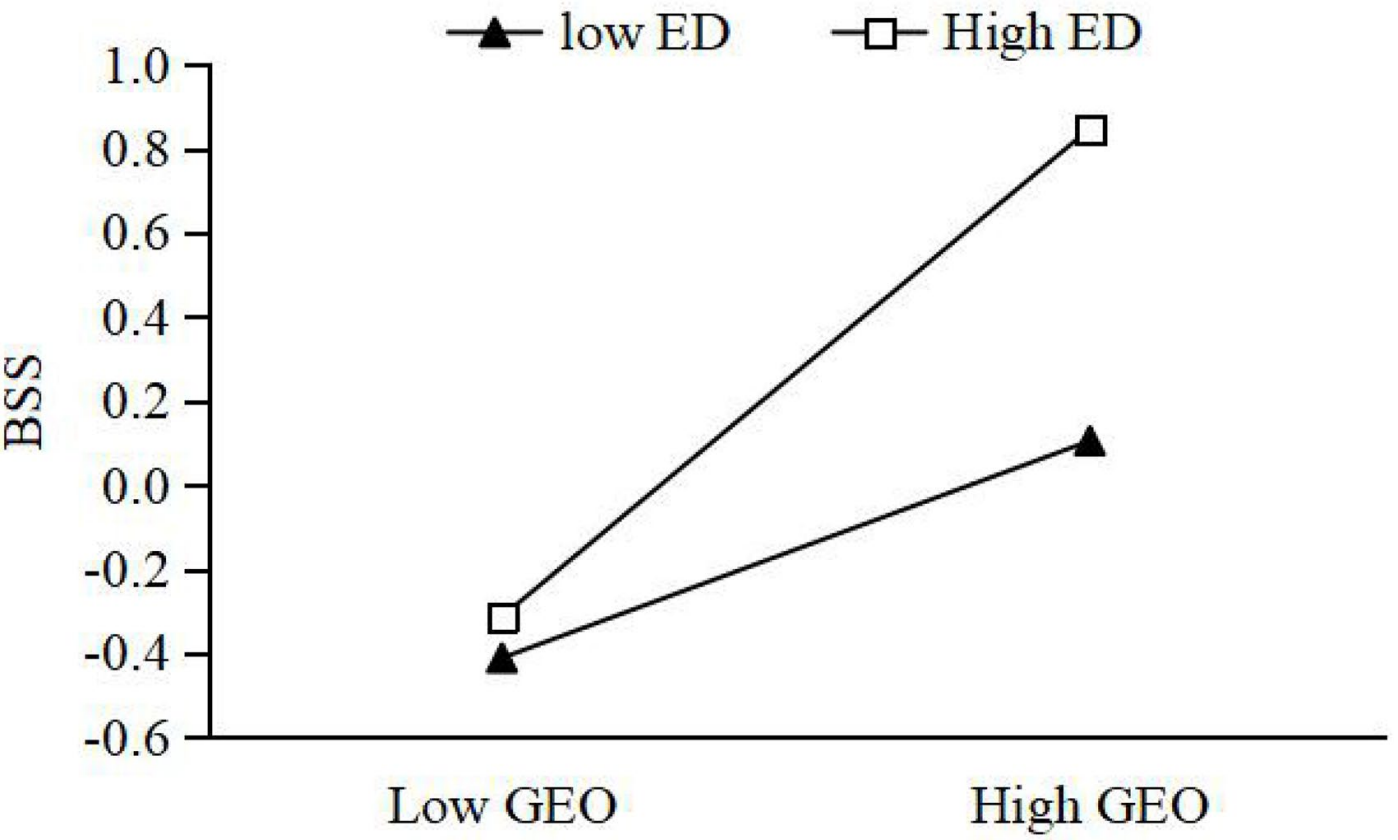
Moderating effect of environmental dynamism (ED) between green entrepreneurial orientation (GEO) and boundary-spanning search (BSS).

**Figure 3:**
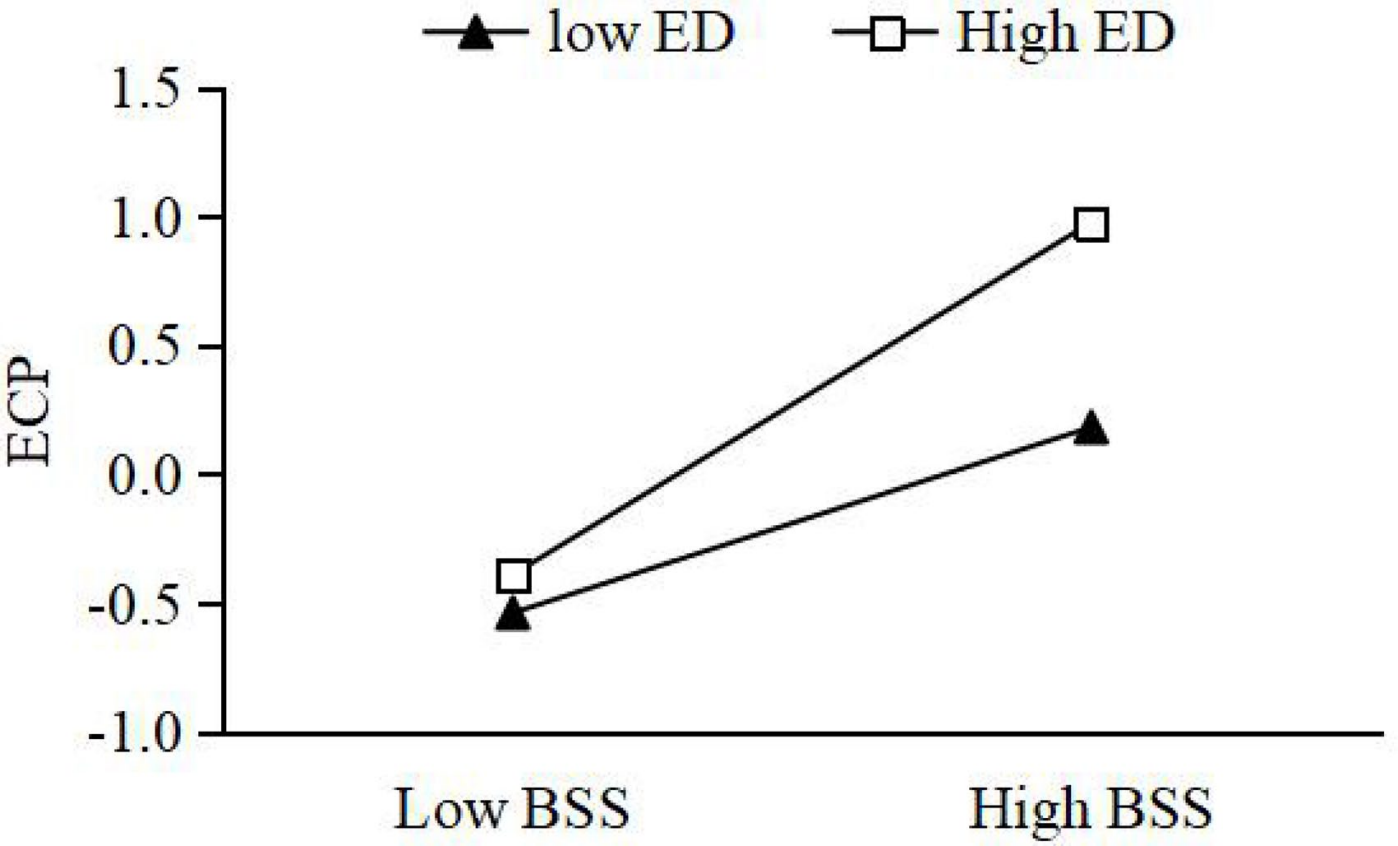
Moderating effect of environmental dynamism (ED) between boundary-spanning search (BSS) and economic performance (ECP).

**Figure 4:**
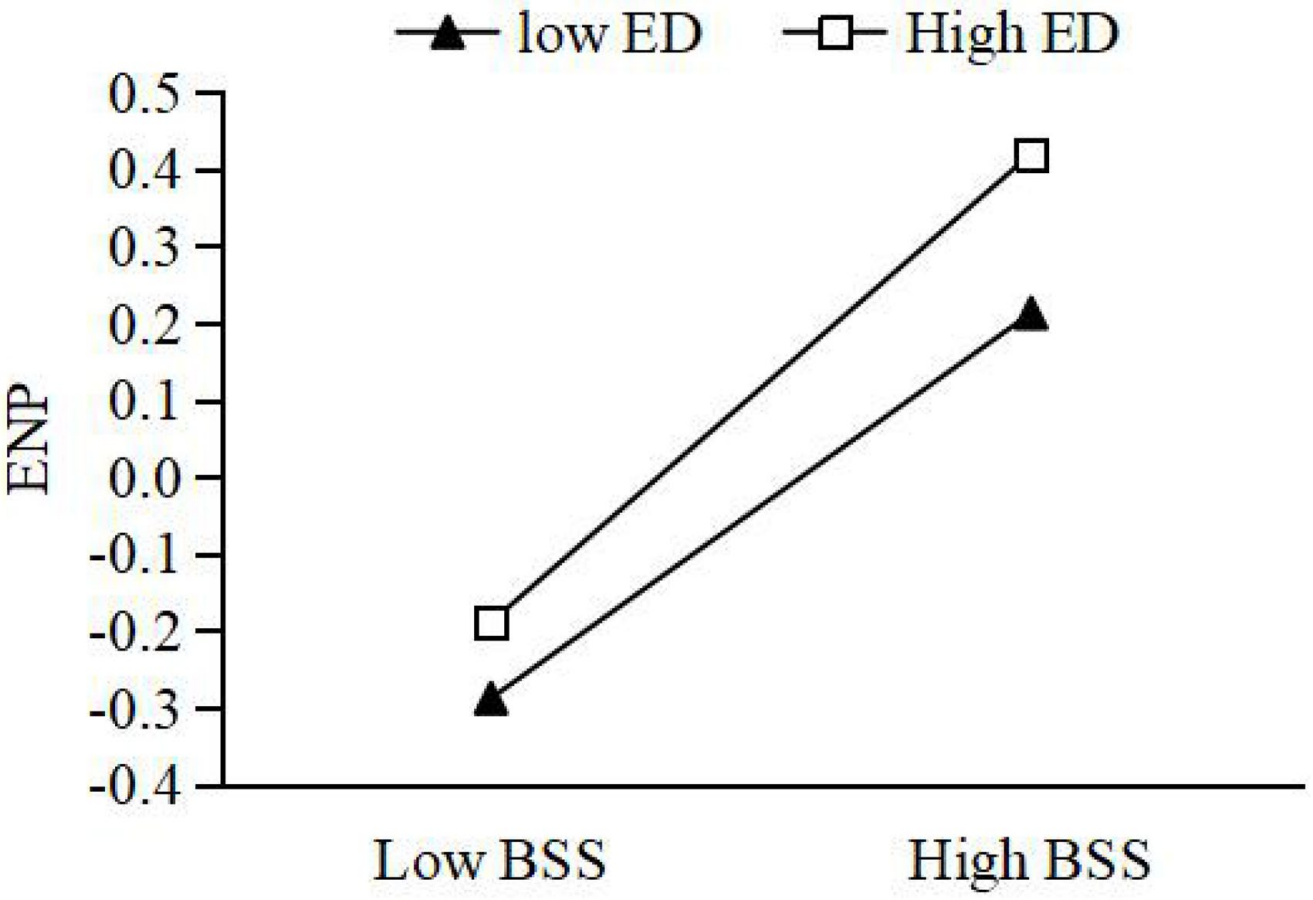
Moderating effect of environmental dynamism (ED) between boundary-spanning search (BSS) and economic performance (ENP).

**Figure 5:**
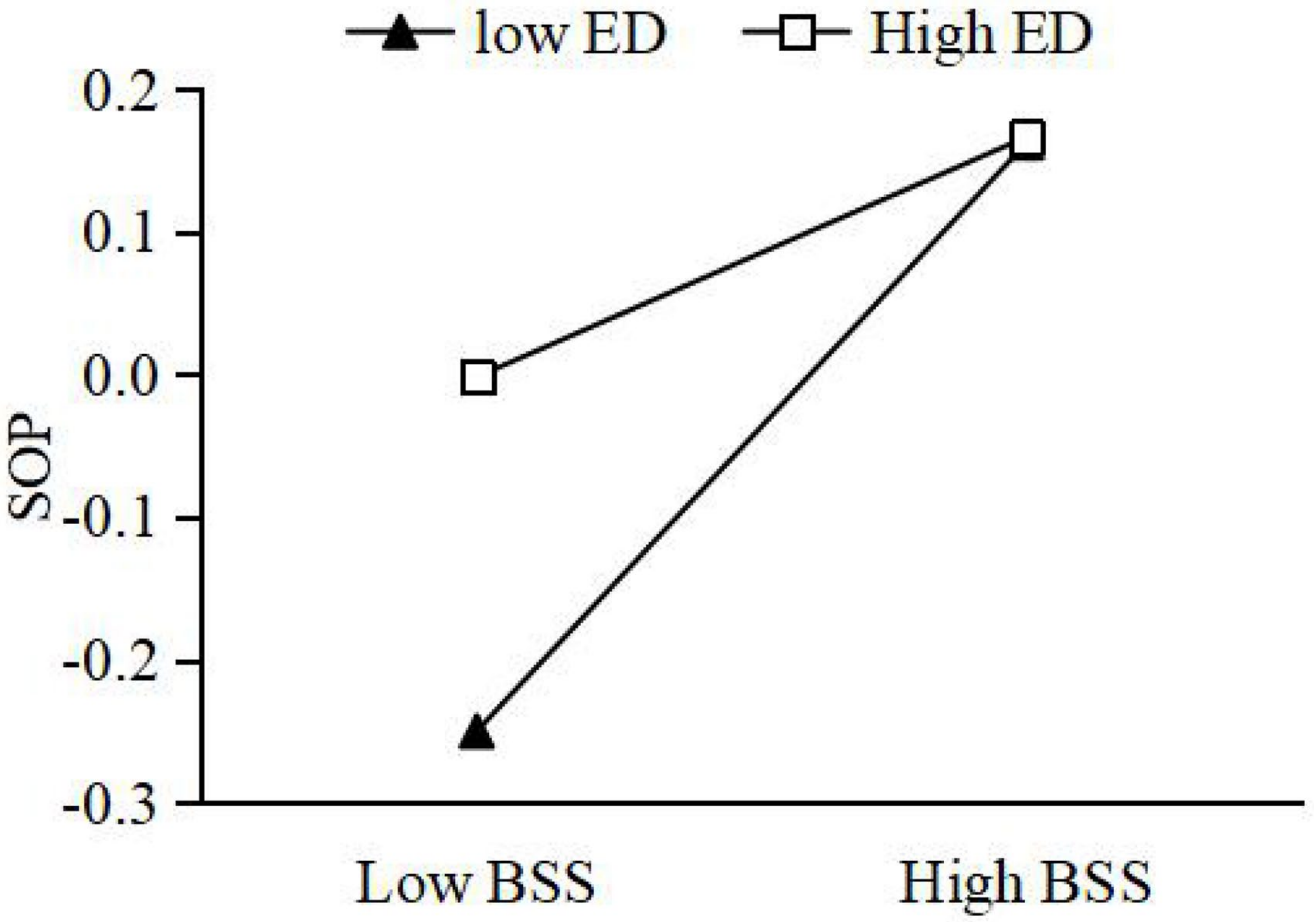
Moderating effect of environmental dynamism (ED) between boundary-spanning search (BSS) and social performance (SOP).

1. Moderating effect of ED between GEO and BSS. On the basis of M2, the interaction term between GEO and ED was added to construct M4. From M4, it can be seen that the interaction term significantly and positively affects BSS (*β* = 0.163, *p* < 0.01). This suggests that ED can reinforce the impact of GEO on BSS. H4 is accepted. Further, this paper takes the ED into M + SD and M − SD, as two high and low ED groups. We used the Process procedure to verify the moderating effect of ED. The results show that under high ED, the simple slope of GEO and BSS is 0.385, with 95% CI [0.034, 0.317], which does not contain 0. Under low ED, the simple slope of GEO and BSS is 0.125, with 95% CI [0.048, 0.219], which does not contain 0. As can be seen from Figure 2, the relationship between GEO and BSS is more significant under high ED, the relationship between GEO and BSS is more significant.
2. Moderating effect of ED between BSS and economic performance. On the basis of M7, the interaction term between BSS and ED is added to construct M9. From M9, it is clear that the interaction term significantly and positively affects economic performance (*β* = 0.119, *p* < 0.01). This suggests that ED reinforces the impact of BSS on economic performance, which is accepted by H5a. This section takes the same approach as above. The results of running the Process program show a simple slope of 0.351, with 95% CI [0.223, 0.519], which does not contain 0, for BSS and economic performance under high ED. Under low ED, the simple slope of BSS and economic performance is 0.178, with 95% CI [0.041, 0.319], which does not contain 0. As shown in Figure 3, the relationship between BSS and economic performance is more significant under high market environments.
3. Moderating effects of ED between BSS and environmental performance. On the basis of M12, the interaction term of BSS and ED is added to construct M14. From M14, the interaction term significantly and positively affects environmental performance (*β* = 0.036, *p* < 0.01), indicating that ED reinforces the effect of cross-border search on environmental performance. H5b is accepted. The results of running the Process procedure show that under high ED, the simple slope of BSS and economic performance is 0.345 with 95% CI [LLCI = 0.159, ULCI = 0.316], which does not contain 0. Under low ED, the simple slope of BSS and economic performance is 0.361 with 95% CI [LLCI = 0.041, ULCI = 0.422], which does not contain 0. As seen in Figure 4, the relationship between BSS search and environmental performance is more significant under high ED.
4. Moderating effects of ED between BSS and social performance. On the basis of M17, the interaction term of BSS and ED is added to construct M19. From M19, the interaction term negatively affects social performance (*β* = −0.104, *p* < 0.01), which indicates that ED weakens the effect of BSS on social performance. H5c is not accepted. Taking the same approach, the results of running the Process program showed a simple slope of 0.148 with 95% CI [−0.013, 0.450] for BSS and social performance under high ED, which does contain 0. The simple slope of BSS and social performance under low ED is 0.365 with 95% CI [0.024, 0.339], which does not contain 0. As shown in Figure 5, the relationship between BSS and social performance is more significant in low market environments.

## Discussion and conclusion

### Conclusion

In the era of environmental protection, influenced by new technologies and changing market dynamics, China in transition, like other developing countries, is in the initial stages of a green turn. Therefore, it has become inevitable for enterprise GEO to explore sustainable development paths. This study constructs a conceptual model for GEO to obtain ESP through BSS, and focuses on the moderating role of ED. Based on this, this study selects 202 manufacturing SMEs in China as the research sample for empirical testing and verifies some of the hypotheses.

Our first finding suggests a positive relationship between GEO and enterprise sustainable economic, environmental and social triad performance. This finding is consistent with previous studies. This suggests that GEO can influence the acquisition of ESP through different mediated transmissions (e.g., Habib et al., 2020). However, little scholarly attention has been paid to the equilibrium state of triadic sustainable performance and the relative state associated with ESP (Koe et al., 2014; Masele, 2019). The results of this study show that although GEO has different explanatory power for economic performance, environmental performance and social performance, the three show a basic balance among them. Together, they play a role in overall ESP.

In a further study, we obtained a second conclusion that there is a full mediating effect of BSS between GEO and triadic sustainable performance of manufacturing SMEs. Although Habib et al. (2020) and Jiang et al. (2018) empirically tested the impact of GEO and the sustainable performance of manufacturing SMEs, the specific behavioral processes of resource acquisition and knowledge combination that translate GEO into ESP were not explored. Starting from organizational search theory, this study takes BSS as an entry point to explain the behavioral process of firms that carry out heterogeneous resource acquisition after establishing a GEO. This elaborates on the green innovative, pioneering, environmental and social characteristics of GEO, which are useful for firms to conduct searches across technological and organizational boundaries. The findings of this study will explain the behavioral process of manufacturing SMEs in the environmental era to obtain triadic sustainable performance of enterprise driven by GEO. The study shows that the four dimensions of GEO clarify the behavioral direction of enterprises’ BSS. The BSS not only enriches the internal resource inventory of enterprises, but also helps to break technological bottlenecks and obtain heterogeneous resources that are difficult to be imitated by competitors, thus winning triadic sustainable performance for enterprises.

In addition, our study finds that ED positively moderates the relationship between GEO and BSS. This study argues that dynamic changes in technology and markets can effectively facilitate the identification of market opportunities in green entrepreneurship orientation. This dynamic change reinforces the ability of GEO to search across borders through positive feedback. Meanwhile, this study also found that ED positively moderates the relationship between BSS with economic performance and environmental performance, but negatively moderates the relationship with social performance. This finding is consistent with existing results on the binary performance of enterprises (Yang et al., 2015; Li and Huang, 2021), but the existing results ignore the positive moderating role of environmental performance in the environmental era in particular. Our findings suggest that there are effective boundaries for triadic sustainable performance in the context of the ED of high technology, markets, and demand for enterprises’ strategies to acquire resources through BSS. In order to survive better in the scenario of the dual environment of government and market regulation, enterprises are more willing to BSS. However, in the search selection enterprises focus on green technologies and environmental management technologies, weakening the social aspect of green entrepreneurship orientation. This leads manufacturing SMEs to focus on economic performance and environmental performance at the expense of socially responsible performance, which adversely affects enterprise sustainable development. This phenomenon is also the plight of the vast majority of developing country firms in the initial stages of the green shift (Shah et al., 2016; Li et al., 2020).

### Practical implications

The findings of this study provide new ideas for manufacturing SMEs to obtain ESP, which has important practical implications.

Firstly, in the era of environmental protection, ESP must be obtained with full consideration of the balance of economic, environmental and social performance. On the one hand, green entrepreneurship means enhancing corporate input. However, manufacturing SMEs are at a competitive disadvantage in terms of resources, so the government should increase its support in policies to stimulate the GEO of enterprises. On the other hand, GEO should bring into play the initiative and pioneer of enterprises in the implementation process, and establish a strategic orientation that takes into account economic, environmental and social aspects (Habib et al., 2021).

Secondly, enterprises should continuously enhance their BSS capabilities and pay attention to the role of BSS on ESP. Boundary-spanning search is a management behavior that aims to acquire heterogeneous resources. This capability has a significant role in searching for internal and external resources across organizational or technological boundaries in manufacturing SMEs. Therefore, on the one hand, enterprises should enhance knowledge learning and absorption to generate green knowledge. This helps to enhance the amount of green-related knowledge savings within the enterprise and achieve green creation. On the other hand, enterprises should also break the confinement of traditional management thinking, enhance boundary-spanning awareness and accelerate BSS activities. Thus, they can break down technological, industry and organizational barriers to build green and sustainable business models that achieve economic, environmental and social triad performance.

Thirdly, under the new environment dynamics, enterprises need to enhance their sensitivity to market opportunity identification, quickly capture the development potential of the dynamic market environment, and promptly adopt GEO to drive BSS, thereby acquiring heterogeneous resources that match the needs of the external environment amidst technological and market uncertainties. Meanwhile, enterprises should stand in the perspective of sustainable development to build a GEO of innovation, pioneer, environmental and social in the dynamic environmental changes. In the process of specific heterogeneous resource acquisition enterprises cannot focus only on current economic and environmental interests and ignore social responsibility, but should focus on the balance and joint growth of economic, environmental and social performance.

### Theoretical contributions

There are three main contributions of this paper. Firstly, this paper introduces the dynamics mechanism of ED into the research framework of organizational search theory. This study explores the role of BSS in the pathway between GEO and enterprise’ triadic sustainable performance by examining the mediating and moderating mechanisms between GEO and ESP, which explains the mechanism “black box” of this causal chain “and theoretical boundaries. Secondly, the research framework of this paper extends the existing “orientation-outcome” to an “orientation-behavior-outcome” research paradigm. This article reveals the process by which green entrepreneurial-oriented firms satisfy heterogeneous resource needs through BSS, which in turn affects the enterprise’ triadic sustainable performance. Thirdly, this paper confirms the performance differences of ED under different action paths. At the same time, this paper integrates GEO, BSS, ED and enterprise’ triadic sustainable performance into the same conceptual model, which is helpful to understand the strategic choice, dual resource acquisition and utilization efficiency of enterprises under the background of ED. It provides some theoretical support for the value co-creation of manufacturing SMEs.

### Limitations and future directions

Although this study examined the transformation and acquisition mechanisms between GEO and ESP at both theoretical and empirical levels, and drew several valuable research findings. However, there are still some research limitations here that warrant further research and exploration in the future.

Firstly, in terms of sustainable performance measurement, the measurement of ESP is still in the exploratory stage due to the small amount of empirical research literature on sustainable performance. Although this study draws on relevant literature, the questionnaire adopted in this study only considers the characteristics of manufacturing SMEs and the perceptions of sustainable performance by enterprise decision-makers. The questionnaire was also modified based on the opinions of experts and managers of manufacturing SMEs. Therefore, the questionnaire used in this paper is only suitable for manufacturing SMEs.

Secondly, in terms of data acquisition, we used a combination of interviews and questionnaires, and try to focus on the representativeness of the variables and indicators, as well as the representativeness of the sample. But questionnaires always have individual differences in perceptions. In the future, we can further adopt data analysis across time stages, selecting corporate reported financial data, environmental information data, and data on social responsibility contributions to represent ESP.

Thirdly, in terms of the research model, this study explores the impact mechanism of GEO on ESP based on a micro-competency perspective. It mainly portrays the individual “work” experience of senior managers. In the future, we can draw on related research to further consider the impact of the behavioral characteristics of the top management team on ESP.

## Data availability statement

The original contributions presented in the study are included in the article/supplementary material, further inquiries can be directed to the corresponding author.

## Author contributions

FY wrote the first draft of the paper and built the theoretical model. YY participated in the writing of the paper. YS and HX participated in surveys. XG organized surveys. JS suggested innovative revisions to the paper. All authors contributed to the article and approved the submitted version.

## Conflict of interest

The authors declare that the research was conducted in the absence of any commercial or financial relationships that could be construed as a potential conflict of interest.

## Publisher’s note

All claims expressed in this article are solely those of the authors and do not necessarily represent those of their affiliated organizations, or those of the publisher, the editors and the reviewers. Any product that may be evaluated in this article, or claim that may be made by its manufacturer, is not guaranteed or endorsed by the publisher.
