# Supplementary material for: Green entrepreneurial orientation, boundary-spanning search and enterprise sustainable performance: The moderating role of environmental dynamism
Source: Front Psychol. 2022 Oct 17;13:978274. doi: 10.3389/fpsyg.2022.978274 (PMC9621121; doi:10.3389/fpsyg.2022.978274)
Supplement: Supplementary file 1 [file Data_Sheet_1.docx]

**Ⅰ Your Basic Information**

| 1. Your gender | 1. male B. female |
| --- | --- |
| 1. Your education background | 1. junior college and below B. bachelor's degree 2. C、master's degree; D、doctorate |
| 1. Time of establishment of your company: | 1. Less than 1 year B. 1-4 years 2. 5-9 years D. More than ten years |
| 1. Your industry: | 1. Agricultural and sideline video processing / Food manufacturing 2. Textile / Clothing / Footwear industry 3. Furniture manufacturing industry \ paper industry \ Entertainment products industry 4. Petrochemical manufacturing industry 5. Medicine / Chemical fiber \ Rubber manufacturing industry 6. Metal non-metallic manufacturing industry 7. Equipment manufacturing / Automobile manufacturing \ Ship manufacturing 8. Electronic \ electrical / instrument manufacturing industry 9. Metal products, machinery and equipment maintenance industry 10. Comprehensive utilization of waste resources |
| 1. Your enterprise size: | 1. Under fifty people B. 50-99 peoples   C. 100-299 peoples D. 300-499 peoples   1. 500-1000 peoples |
| 1. Your position in the company: | 1. Executives B. Senior executives;   C. Assistants D. General managers   1. Executive Directors/Directors/CEOs |
| 1. You manage position time: | 1. Less than 1 year B. 1-4 years   C. 5-9 years D. More than ten years |
| 1. Your company's annual sale | 1. 1 million the following B. 1 million - 4.99 million   C. 5 million - 9.99 million D. 10 million - 49.99 million   1. 50 million - 99.99 million F. 100 million - 400 million |

**Ⅱ Below are the relevant questions about green entrepreneurship of SMEs. Please tick "√"according to your actual situation and real idea.**

**1. As a business executive, what do you know about the following events:**

| NO. | The problem | Strongly Disagree | Disagree | General | Agree | Strongly Agree |
| --- | --- | --- | --- | --- | --- | --- |
| 1 | Your company's green transformation of existing products or production lines is relatively strong | ① | ② | ③ | ④ | ⑤ |
| 2 | Your company has developed new green products or production lines with a great degree | ① | ② | ③ | ④ | ⑤ |
| 3 | Your company emphasizes green product or production innovation, pursues technological leadership, and has outstanding research and development activities | ① | ② | ③ | ④ | ⑤ |
| 4 | Your company is committed to improving its overall environmental awareness | ① | ② | ③ | ④ | ⑤ |
| 5 | Your company is very concerned about the impact of business activities on environmental protection | ① | ② | ③ | ④ | ⑤ |
| 6 | Your company will invest funds to control the impact of its own operations on the environment | ① | ② | ③ | ④ | ⑤ |
| 7 | Your company usually takes green action first, and competitors follow up or respond later | ① | ② | ③ | ④ | ⑤ |
| 8 | Senior executives constantly review industry trends, seize market opportunities and respond to market changes in advance | ① | ② | ③ | ④ | ⑤ |
| 9 | Your company often launches green new products ahead of its competitors, and tends to be the "leader" in new markets, or the first to introduce new management models, new green technologies, and product and service innovations | ① | ② | ③ | ④ | ⑤ |
| 10 | Your company actively assumes corporate social responsibility on environment | ① | ② | ③ | ④ | ⑤ |
| 11 | Your company's positive actions affect the public's green behaviors | ① | ② | ③ | ④ | ⑤ |
| 12 | Your company actively participates in local green development construction | ① | ② | ③ | ④ | ⑤ |

**2. As a business manager, how do you collect resources and knowledge for your company?**

| **NO.** | **The problem** | **Strongly Disagree** | **Disagree** | **General** | **Agree** | **Strongly Agree** |
| --- | --- | --- | --- | --- | --- | --- |
| **When learning from peers and non-peers about new technologies that are different from existing technology systems, your business aims to** | | | | | | |
| 1 | Lead competitors in identifying new opportunities in new markets and new customer groups | ① | ② | ③ | ④ | ⑤ |
| 2 | Develop new ways to meet customer needs ahead of competitors | ① | ② | ③ | ④ | ⑤ |
| 3 | Develop product features that competitors don't have | ① | ② | ③ | ④ | ⑤ |
| 4 | Develop new processes for product development that competitors do not have | ① | ② | ③ | ④ | ⑤ |
| **When learning from peers and non-peers about new technologies that are different from existing technology systems, your business aims to** | | | | | | |
| 5 | Strengthen the competitive position of the company's existing products in the market | ① | ② | ③ | ④ | ⑤ |
| 6 | Follow competitors to improve the quality of existing products | ① | ② | ③ | ④ | ⑤ |
| 7 | Follow competitors to improve the productivity of existing products | ① | ② | ③ | ④ | ⑤ |
| 8 | Follow competitors to improve the existing product development process | ① | ② | ③ | ④ | ⑤ |

**3.As a business executive, how do you feel about the extent to which the company’s internal and external environments** **change**

| **NO.** | **The problem** | **Strongly Disagree** | **Disagree** | **General** | **Agree** | **Strongly Agree** |
| --- | --- | --- | --- | --- | --- | --- |
| 1 | Competitors in this industry are predictable | ① | ② | ③ | ④ | ⑤ |
| 2 | It's hard to make a product or service obsolete | ① | ② | ③ | ④ | ⑤ |
| 3 | The industry's customer needs or preferences are easy to predict | ① | ② | ③ | ④ | ⑤ |
| 4 | The technology of production in this industry changes slowly | ① | ② | ③ | ④ | ⑤ |
| 5 | Economic, technological, social, or political changes are easy to predict | ① | ② | ③ | ④ | ⑤ |

**4. Rate the extent to which your firm has made an improvement in its performance based on environmental management practice adoption.**

| NO. | The problem | Strongly Disagree | Disagree | General | Agree | Strongly Agree |
| --- | --- | --- | --- | --- | --- | --- |
| 1 | Your company's material purchasing costs have gone down. | ① | ② | ③ | ④ | ⑤ |
| 2 | Your company's energy cost have been reduced. | ① | ② | ③ | ④ | ⑤ |
| 3 | Your company's waste disposal fees have been decreased. | ① | ② | ③ | ④ | ⑤ |
| 4 | Your company's return on investment has been improved | ① | ② | ③ | ④ | ⑤ |
| 5 | Your company's earnings continue to be improved | ① | ② | ③ | ④ | ⑤ |
| 6 | Your company has reduced air emissions | ① | ② | ③ | ④ | ⑤ |
| 7 | Your company has reduced waste (water and/or solids) emissions | ① | ② | ③ | ④ | ⑤ |
| 8 | Your company has reduced the consumption of dangerous/harmful/toxic substances | ① | ② | ③ | ④ | ⑤ |
| 9 | Your company has lowered the frequency of environmental accidents | ① | ② | ③ | ④ | ⑤ |
| 10 | Your company has saved energy and improved efficiency | ① | ② | ③ | ④ | ⑤ |
| 11 | Your company has improved or bettered the welfare of its overall stakeholders | ① | ② | ③ | ④ | ⑤ |
| 12 | Your company has helped to improve health and safety in your area | ① | ② | ③ | ④ | ⑤ |
| 13 | Your company has reduced the environmental impact and risk to the general public | ① | ② | ③ | ④ | ⑤ |
| 14 | Your company has improved the occupational health and safety of employees | ① | ② | ③ | ④ | ⑤ |
| 15 | Your company has Improved its awareness and protection of the claims and rights of people serving in community | ① | ② | ③ | ④ | ⑤ |
